# Vascular endothelial growth factor-B gene transfer exacerbates retinal and choroidal neovascularization and vasopermeability without promoting inflammation

**Published:** 2011-02-17

**Authors:** Xiufeng Zhong, Hu Huang, Jikui Shen, Serena Zacchigna, Lorena Zentilin, Mauro Giacca, Stanley A. Vinores

**Affiliations:** 1 Wilmer Eye Institute, Johns Hopkins University School of Medicine, Baltimore, MD; 2 Molecular Medicine Laboratory, International Centre for Genetic Engineering and Biotechnology (ICGEB), Padriciano, Trieste, Italy

## Abstract

**Purpose:**

The role of vascular endothelial growth factor (VEGF)-B in the eye is poorly understood. The present study was conducted to evaluate the effect of overexpression of VEGF-B via adeno-associated virus (AAV) gene transfer on ocular angiogenesis, inflammation, and the blood-retinal barrier (BRB).

**Methods:**

Three recombinant AAV vectors were prepared, expressing the 167 (*AAV-VEGF-B167*) or 186 amino acid isoform (*AAV-VEGF-B186*) of VEGF-B or the green fluorescent protein (*GFP*) reporter gene (*AAV-GFP*). Approximately 1×10^9^ viral genome copies of *AAV-VEGF-B167*, *AAV-VEGF-B186*, or *AAV-GFP* were intraocularly injected. The efficacy of the gene transfer was assessed by directly observing GFP, by immunohistochemistry, or by real-time PCR. A leukostasis assay using fluorescein isothiocyanate-conjugated Concanavalin A was used to evaluate inflammation. The BRB was assessed using a quantitative assay with ^3^H-mannitol as a tracer. Retinal neovascularization (NV) was assessed at postnatal day 17 in oxygen-induced ischemic retinopathy after intravitreal injection of *AAV-VEGF-B* in left eyes and *AAV-GFP* in right eyes at postnatal day 7. Two weeks after injection of AAV vectors, choroidal NV was generated by laser photocoagulation and assessed 2 weeks later.

**Results:**

GFP expression was clearly demonstrated, primarily in the retinal pigment epithelium (RPE) and outer retina, 1–6 weeks after delivery. mRNA expression levels of *VEGF-B167* and *VEGF-B186* were 5.8 and 12 fold higher in the *AAV-VEGF-B167*- and *AAV-VEGF-B186*-treated groups, respectively. There was no evidence of an inflammatory response or vessel abnormality following injection of the vectors in normal mice; however, VEGF-B increased retinal and choroidal neovascularization. *AAV-VEGF-B186*, but not *AAV-VEGF-B167*, enhanced retinal vascular permeability.

**Conclusions:**

VEGF-B overexpression promoted pathological retinal and choroidal NV and BRB breakdown without causing inflammation, which is associated with the progression of diabetic retinopathy and age-related macular degeneration, showing that these complications are not dependent on inflammation. VEGF-B targeting could benefit antiangiogenic therapy.

## Introduction

Members of the vascular endothelial growth factor (VEGF) family have been identified as key molecules in the development of many of the adverse effects of retinopathy of prematurity (ROP), diabetic retinopathy (DR), age-related macular degeneration (AMD), and other ischemic and inflammatory retinopathies. The complications that are attributable, at least in part, to VEGF include retinal and choroidal neovascularization (CNV) and other vascular abnormalities, blood-retinal barrier (BRB) breakdown, and increased leukostasis.

Three members of the VEGF receptor family have been identified: VEGFR1, 2, and 3 [1]. Each of these distinct tyrosine kinase receptors binds only certain VEGF family members. Ligands of VEGFR1 include VEGF-A, VEGF-B, and placental growth factor (PlGF); ligands of VEGFR2 include VEGF-A, VEGF-C, and VEGF-D; and the ligands of VEGFR3 are VEGF-C and VEGF-D; thus, PlGF and VEGF-B uniquely bind VEGFR1, and VEGF-E uniquely binds VEGFR2 [2-6]. In human and monkey retinas, all three VEGF receptors are expressed in nonvascular areas, but only VEGFR1 is constitutively expressed in retinal microvessels, predominantly on pericytes [2,7-10].

There is some confusion regarding the role of VEGF in BRB breakdown and neovascularization (NV) in ischemic retinopathies. There is strong evidence suggesting that VEGF is the primary cytokine causing vascular leakage and NV in ischemic retinas [2,11-15], but there is mRNA and protein expression of VEGF-A, VEGFR1, and VEGFR2 in normal retinas, suggesting physiologic functions for VEGF [2,16-19]. VEGF is a secreted protein, so it may have paracrine, as well as autocrine effects [20]. VEGFR1 is believed to function in inflammation [21], but there is also convincing evidence that angiogenesis and vascular permeability are regulated, directly or indirectly, through VEGFR1 [22]. Most of the biologic functions attributed to VEGF-A appear to be mediated through VEGFR2, with the roles of the other receptor types being less clear [2,7,23]. The function of VEGFR1 remains controversial, but there is evidence that it can function as a negative regulator of VEGFR2 [24]. Soluble forms of VEGFR1 and R2 also exist, and some of these splice variants have specificities different from those of the membrane-bound forms [25-27].

PlGF is a member of the VEGF family that binds to VEGFR1, but not VEGFR2. This factor can stimulate pathological, but not physiologic angiogenesis [28-32], and the migration and proliferation of endothelial cells [28]. It potentiates the effect of VEGF on vascular permeability [9,30,33,34], and it can induce chemotaxis of monocytes, which express VEGFR1, but not VEGFR2 [22,29,34-36]. These findings show that PlGF plays a role in angiogenesis and inflammation, both of which are hallmarks of DR and AMD, but unlike VEGF, PlGF is not upregulated by hypoxia [37]. Prenatal vascular development in mice is unaffected by a PlGF deficiency. However, our previous findings demonstrate that anti-PlGF treatment inhibits retinal and choroidal NV, BRB breakdown, and inflammation in experimental models [22], providing support for the possibility that PlGF, through its binding to VEGFR1, participates in the development of these complications of ocular disorders. A deficiency in PlGF or the neutralization of VEGFR1 suppressed laser-induced CNV, reduced inflammation, and reduced retinal NV in oxygen-induced ischemic retinopathy (OIR) [29,30,38], as did deletion of the hypoxia response element from the *VEGF* promoter [39]. It is not clear whether PlGF stimulates pathological angiogenesis and vascular permeability by signaling through VEGFR1 or by displacing VEGF-A from VEGFR1, thereby increasing the fraction available to activate VEGFR2 [9]. Nonetheless, depletion of PlGF clearly inhibits these complications in experimental models of ocular disease.

Despite its early discovery and high sequence homology to the other VEGF family members, the biologic function of VEGF-B remained debatable for a long time. VEGF-B, like PlGF, binds to VEGFR1, but not VEGFR2. It has a wide tissue distribution, with the greatest abundance in the heart, skeletal muscles, and diaphragm [40,41]; however, the *VEGF-B* promoter lacks a hypoxia response element, so it is not induced by hypoxia as VEGF-A is [33]. Neither VEGF-B nor PlGF were able to compensate for VEGF-A during its blockade, and mice lacking either factor displayed only minor developmental defects [42]. VEGF-B knockout mice were viable and fertile, but had subtle cardiac abnormalities [43,44]. There are two isoforms of VEGF-B (VEGF-B167 and VEGF-B186) due to alternative splicing [45,46]. The predominant isoform, VEGF-B167, which is four times more abundant than VEGF-B186, has a COOH-terminal heparin-binding domain, allowing it to bind to pericellular heparin-like glycosaminoglycans, thus anchoring it to the extracellular matrix [46].

Contrasting results were obtained by analyzing the angiogenic effects of VEGF-B in normal and pathologic conditions, with some studies showing that VEGF-B is angiogenic [47-50] and others reporting that it is not [42,44,51-53]. In particular, VEGF-B167 was reported to have significantly increased revascularization of the infarcted myocardium; however, it failed to enhance vascular growth in the skin or ischemic limb [54]. Another recent study, involving the delivery of VEGF-B186 after infarction in pigs and rabbits, indicated that the factor induced myocardial-specific angiogenesis and arteriogenesis [55]. This study, however, used gene transfer with adenoviral vectors, which notoriously induce inflammation and an immune response. Finally, gene delivery of VEGF-B167 to the heart using adeno-associated vectors (AAVs) has shown that the factor has a marked beneficial effect after myocardial infarction in rats [56] or pacing-induced heart failure in dogs [57], in the absence of a significant angiogenic effect. Another recent study showed that VEGF-B was not essential for the growth of new vessels, but it was critical for their survival [58], affecting endothelial cells, pericytes, smooth muscle cells, and vascular progenitor cells. Through the use of AAV vectors, the present study was conducted to investigate the effect of continuous expression of both isoforms of VEGF-B directly on the retina.

## Methods

### Production, purification, and characterization of recombinant adeno-associated virus vectors

Three recombinant AAV vectors expressing green fluorescent protein (GFP) and the 167 and 186 amino acid isoforms of mouse VEGF-B (*AAV-GFP*, *AAV-VEGF-B167*, and *AAV-VEGF-B186*, respectively) were prepared by the AAV Vector Unit at ICGEB Trieste. All the vectors were pseudotyped following a cross-packaging approach whereby the AAV type 2 vector genome was packaged into AAV capsid serotype 9 [59,60]. Methods for production and purification were previously described [61,62]. Briefly, viral vector stocks were obtained in HEK293 cells by high-scale calcium phosphate co-precipitation of the AAV backbone plasmid along with helper plasmids, expressing the AAV genes coding for the replicative and capsid proteins and the adenovirus helper functions supporting AAV replication. Viral vector particles were then purified from crude cell lysates by cesium chloride gradient ultracentrifugation followed by dialysis of pooled fractions. Among the different serotypes that were used for retinal gene delivery, AAV serotype 9 (AAV9) showed robust and widespread transduction in the outer retina, inner retina, retinal ganglion cell (RGC) layers [63,64], and synaptic layers [65,66]. Based on these results, we decided to develop an AAV9-based vector for VEGF-B expression in the retina in our experimental settings. The vectors used in this study express the exogenous gene under the control of the strong, constitutive, immediate early promoter from the human cytomegalovirus (CMV).

### Mice for gene transduction studies

Pathogen-free C57BL/6 mice from Charles River Laboratories (Wilmington, MA) were treated in accordance with the Association for Research in Vision and Ophthalmology Statement for the Use of Animals in Ophthalmic and Vision Research, the guidelines of the Johns Hopkins University Animal Care and Use Committee, and the guidelines of the Institute for Laboratory Animal Research (Guide for the Care and Use of Laboratory Animals).

### Intraocular injections of adeno-associated virus vectors in mice

Subretinal or intravitreous injections were administered with a Harvard pump microinjection apparatus and pulled glass micropipets, as previously described [67]. Briefly, under a dissecting microscope, the sharpened tip of a micropipette was passed through the sclera, just behind the limbus into the vitreous cavity. Each micropipet was calibrated to deliver 1 μl of vehicle containing approximately 1×10^9^ viral genome copies (vgc) of *AAV-VEGF-B167*, *AAV-VEGF-B186*, or *AAV-GFP*, upon depression of a foot switch.

### Real-time PCR

Total RNA was isolated from retinas using the RNeasy Mini Kit (Qiagen, Alameda, CA), and RNA quality was measured using a NanoDrop1000 spectrophotometer (Thermo Scientific, Waltham, MA). The cDNA was prepared using the SuperScript First-Strand Synthesis System (Invitrogen, Carlsbad, CA), then used as a template for real-time PCR amplification to detect the expression levels of murine VEGF-B167 and 186. Real-time PCR was performed using iQ SYBR Green Supermix and MyiQ Single-Color Real-Time PCR Detection System (BioRad, Hercules, CA). The forward and reverse primers that were used are shown in Table 1. All primers were synthesized by Integrated DNA Technologies (Coralville, IA). Five serial eightfold dilutions of one of the samples were used to establish a standard curve in each experiment with the primer *Actb *(β-actin). The expression level of each gene was normalized to that of *Actb*. Samples were prepared in at least triplicate, and real-time PCR measurement of each sample was done in triplicate. The formation of a single product was also confirmed by observing the melting-curve graph that was generated by the thermal cycler machine, MyiQ, for each reaction tube. Agarose gel electrophoresis was used to confirm that reaction products had the expected size.

**Table 1:** Primers used for Real-time PCR

**Genebank**	**Primers**	**Forward**	**Reverse**	**Size (bp)**	**Species**
NM_011697	VEGF-B167	CCTGGAAGAACACAGCCAAT	GGAGTGGGATGGATGATGTC	164	mouse
NM_011697.2	VEGF-B186	CCAGACAGGGTTGCCATA	GCTGGAGTGGGATGGATG	110	mouse
NM_007393.2	β-actin	GCCTTCCTTCTTGGGTATGG	GCAATGATCTTGATCTTCATGG	204	mouse

### Fluorescence microscopy after subretinal injection of *AAV-GFP*

To verify the expression of AAV vectors in retinas, 1 to 6 weeks after subretinal injection of the *AAV-GFP* vector or phosphate buffered saline (PBS; 137 mM NaCl, 2.7 mM KCl, 8 mM Na_2_HPO_4_, and 2 mM KH_2_PO_4_), mice were humanely sacrificed and eyes were rapidly removed and immersed in 10% formalin for at least 3 h at room temperature. Some eyes were used to make choroidal or retinal flatmounts, the others for preparing cryosections. To prepare retinal flatmounts, the anterior segments of eyes were removed and the entire retina was carefully dissected from the remaining eyecups. Radial cuts were made from the edge to the equator of the retina, and the retina was flatmounted in aquamount (Thermo Fisher Scientific, Waltham, MA) with the photoreceptors facing down. For choroidal flatmounts, radial cuts were also made in the remaining eyecups, which were flatmounted with the sclera facing down. To prepare cryosections, the cornea and lens were removed and the eyecups were cryoprotected in 20% sucrose in PBS overnight at 4 °C. Eyes were then embedded in optimal cutting temperature embedding compound (OCT; Miles Diagnostics, Elkhart, IN) and frozen in a slurry of dry ice and isopentane. Ten-micron frozen sections were cut, then dried in air for 20 min, rinsed in PBS, and mounted with aquamount containing 0.2 μg/ml 4´, 6-diamidino-2´-phenylindole, dihydrochloride. Flatmounts and sections were examined using fluorescence microscopy (Axioskop microscope; Zeiss, Thornwood, NY), and images were digitized using a three-color charge-coupled device video camera (IK-TU40A; Toshiba, Tokyo, Japan) and a frame grabber.

### Immunofluorescence staining for vascular endothelial growth factor-B

Mice treated with a subretinal injection were humanely killed after 4 weeks, and the eyes were removed and fixed with 4% paraformaldehyde in PBS for 15 min. Then, the anterior segments were removed, and the remaining posterior retina was immersed again in a 4% paraformaldehyde solution at 4 °C overnight. Eyes were sequentially washed with PBS and 12.5% and 25% sucrose, and then embedded in OCT. Ten-micron frozen sections were cut, air dried, and washed with PBS. Specimens were blocked with 10% normal goat serum in PBS for 30 min at room temperature to prevent nonspecific binding. The slides were incubated with a 1:50 dilution of mouse monoclonal antihuman/antimouse VEGF-B antibody (MAB751; R&D), which identifies both VEGF-B isoforms, in 2% normal goat serum /PBS overnight at 4 °C in a humidified chamber, then washed with PBS three times, 5 min each time, and incubated with Alexa Fluor 594 goat antimouse IgG (H^+^L) secondary antibody (Molecular Probes, Carlsbad, CA) for 1.5 h at 37 °C. After washing, the slides were mounted with aquamount containing 0.2 μg/ml 4´, 6-diamidino-2´-phenylindole, dihydrochloride. For a parallel negative control, the primary antibody was omitted and PBS was substituted. Sections were examined using fluorescence microscopy (Axioskop microscope; Zeiss) and confocal microscopy (Zeiss LSM 710), and images were taken under the same conditions for optimal comparison.

### Histological examination

Four weeks after vector injections into 4-week-old normal C57BL/6J mice, mice from each group were euthanized. Cryosections were prepared as described above. Eyes were serially sectioned at 10 μm thickness, mounted onto Superfrost/Plus microscope slides (Fisher Scientific, Pittsburgh, PA), stained with hematoxylin and eosin, and examined by light microscopy.

### Quantitative measurement of leukostasis

Leukocytes were labeled with fluorescein isothiocyanate (FITC)-conjugated Concanavalin A (Vector Labs, Burlingame, CA) as previously described [68]. Briefly, mice were perfused with PBS to remove erythrocytes and non-adherent leukocytes, followed by a perfusion with fluorescein-conjugated concanavalin A to label leukocytes. Another PBS perfusion was used to flush out unbound fluorescein. Retinal flatmounts were prepared and examined with the Axioskop microscope. The total numbers of leukocytes adhering to the retinal vessels were counted at 200× by the same investigator, with the investigator being masked as to the nature of the specimen.

### Quantitative assessment of the blood–retinal barrier

Quantitative measurement of the breakdown of the BRB was performed as previously described [68,69]. Briefly, mice subretinally injected with *AAV-VEGF-B167*, *AAV-VEGF-B186*, or *AAV-GFP* control vector were sedated and given an intraperitoneal injection of 1 μCi/g bodyweight of [^3^H]-mannitol after 4 weeks. One hour after injection, the mice were sedated, retinas were rapidly removed and dissected to free them from the lens, vitreous, and any retinal pigment epithelium (RPE) that has been extruded. The retinas were then placed into preweighed scintillation vials. The thoracic cavity was opened, and the left superior lobe of the lung was removed, blotted to remove excess blood, and placed in another preweighed scintillation vial. A left dorsal incision was made, and the retroperitoneal space was entered without entering the peritoneal cavity. The renal vessels were clamped with a forceps, and the left kidney was removed, cleaned of all fat, blotted, and placed into a preweighed scintillation vial. The remaining droplets on the tissues were allowed to evaporate for 20 min. The vials were weighed, and the tissue weights were calculated and recorded. One ml of NCSII solubilizing solution was added, and the vials were incubated overnight in a 50 °C water bath. The solubilized tissue was brought to room temperature and decolorized with 20% benzoyl peroxide in toluene in a 50 °C water bath. After re-equilibrating it to room temperature, 5 ml of Cytoscint ES (Fisher Scientific) and 30 μl of glacial acetic acid (Sigma, St. Louis, MO) were added, and the vials were stored for several hours in darkness at 4 °C to eliminate chemoluminescence. Radioactivity was counted with a Wallac 1409 Liquid Scintillation Counter. The CPM/mg tissue measured for the lungs, kidneys, and retinas from the experimental and control groups was used to calculate the retina-to-lung (RLLR) and retina-to-renal leakage ratios (RRLR). The ratios obtained for retinas treated with *AAV-VEGF-B167* or *AAV-VEGF-B186* were compared with those treated with *AAV-GFP* control vectors by Student’s unpaired *t* test for populations with unequal variances.

### Animal model of oxygen-induced ischemic retinopathy

Postnatal day 7 (P7), C57BL/6J mice were given an intravitreal injection of 1 μl of vehicle containing approximately 1×10^9^ vgc of *AAV-GFP*, *AAV-VEGF-B167*, or *AAV-VEGF-B186* and placed in a 75% oxygen box for 5 days. At P12, the mice were returned to room air. At P17, the mice were humanely sacrificed. Eyes were enucleated and fixed in 4% paraformaldehyde for 30 min at room temperature. Retinas were dissected and stained overnight at room temperature with fluoresceinated Griffonia Simplicifolia Isolectin B4 (Alexa Fluor 594–conjugated I21413; 1:100 dilution; Molecular Probes) in PBS. Following three washes, the retinas were whole-mounted onto Superfrost/ Plus microscope slides with the photoreceptor side down.

Quantification of retinal NV was performed as described previously [70,71]. Briefly, images of each of the four quadrants of whole mounted retinas were taken at 5× magnification on a Zeiss Axioplan 2 microscope and imported into Adobe Photoshop (Adobe Systems, Inc., San Jose, CA). Retinal segments were merged to produce an image of the entire retina. Neovascular tuft formation was quantified by comparing the number of pixels in the affected areas with the total number of pixels in the retina. Percentages of NV from mouse retinas treated with *AAV-VEGF-B167* or *186* were compared with those from the retinas treated with *AAV-GFP*. Measurement of retinal NV was done blind to the identity of the sample.

### Animal model of laser-induced choroidal neovascularization

Two weeks after vector injection, Bruch’s membrane was ruptured by laser photocoagulation at three locations in each eye as previously described [72]. Briefly, C57BL/6 mice were anesthetized with ketamine hydrochloride (100 mg/kg bodyweight), and their pupils were dilated. Laser photocoagulation (75 μm spot size, 0.1 s duration, 120 mW) was performed in the 9, 12, and 3 o’clock positions of the posterior pole of each eye with the slit-lamp delivery system of an OcuLight GL diode laser (Iridex, Mountain View, CA), and a handheld coverslip was used as a contact lens to view the retina. Production of a tissue bubble by the laser, which indicates ruptures of Bruch’s membrane, is an important factor in obtaining CNV; therefore, only burns in which a bubble was produced were included in the study.

After 2 weeks, the mice were perfused with 1 ml of PBS containing 50 mg/ml of fluorescein-labeled dextran (2×10^6^ Da average molecular mass; Sigma-Aldrich, St. Louis, MO), and choroidal flatmounts were examined by fluorescence microscopy. Images were captured at 20× magnification on a Zeiss Axioplan 2 microscope. Image analysis software (Image-Pro Plus; Media Cybernetics, Silver Spring, MD) was used to measure the total area of CNV at each rupture site, with the investigator masked with respect to treatment group. This is a widely used and accepted method for assessing experimental CNV [72,73].

### Statistical analysis to determine significance

Data were expressed as mean±SEM. Statistical analysis was performed using Student’s *t* test; p<0.05 was considered significant.

## Results

### Construction and characterization of recombinant adeno-associated virus vectors

**Figure 1:**
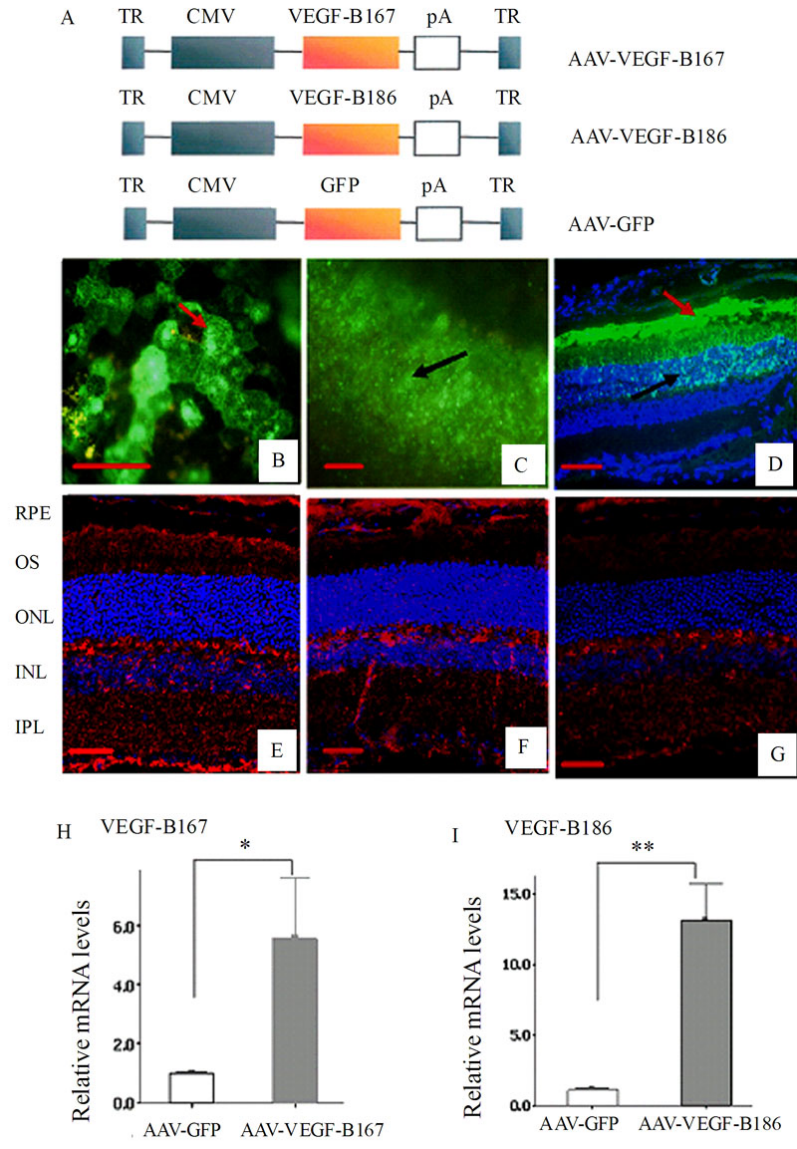
This figure shows the transgene expression of recombinant adeno-associated virus (rAAV) vectors in the retinas of 4-week-old C57BL/6J mice, 4 weeks after subretinal injection of approximately 1×10^9^ vgc recombinant adeno-associated virus vectors. **A**: This shows the schematic representation of the three rAAV vectors used in this study for transducing vascular endothelial growth factor (*VEGF*)-*B167*, *VEGF-B186,* and the marker *GFP* gene. Terminal repeats (TR), AAV terminal repeats; cytomegalovirus (CMV), human cytomegalovirus immediate–early promoter; pA, polyadenylation site. **B**–**D**: Expression of GFP protein is demonstrated in flatmounts of the choroid (**B**), retina (**C**), and a section of eye (**D**); note the greatest expression of green fluorescent protein (GFP) in the retinal pigment epithelium (RPE; **B** and **D**, red arrow) and the outer retina (**C** and **D**, black arrow). **E**–**G**: Immunofluorescence staining indicated that the VEGF-B167 (**E**) and VEGF-B186 proteins (**F**) are more strongly expressed in retinas from experimental groups—mainly in the RPE—than in retinas from the GFP control group (**G**). Cell nuclei were stained with 4´, 6-diamidino-2´-phenylindole dihydrochloride. Images **E**–**G** were taken under the same conditions for optimal comparison. **H**–**I**: Real-time PCR analysis of VEGF-B167 (**H**) and 186 (**I**) expression shows that mRNA expression levels of VEGF-B167 and 186 in the *AAV-VEGF-B167* and *186* groups are significantly higher than those in the *AAV-GFP* control group (in arbitrary units normalized against mouse β-actin; *p<0.05, **p<0.001). Data are espressed ±SEM and sample sizes were 3. **B**–**G**: Scale bar represents 50 µm.

Three vectors based on the AAV serotype 9 (AAV9) were constructed, respectively, expressing the 167 and 186 amino acid isoforms of mouse VEGF-B (*AAV-VEGF-B167* and *AAV-VEGF-B186*) or the green fluorescence protein gene (*AAV-GFP*) under the control of the human CMV promoter (Figure 1A). For the three vector preparations, titers were approximately 1.0×10^12^ vgc/ml.

### Transgene expression of recombinant adeno-associated virus vectors in the mouse retina

To determine the location and the time course of rAAV expression in mouse retinas, 4-week-old C57BL/6J mice received subretinal injections of *AAV-GFP* or PBS. Animals were killed 1–6 weeks after injection. Under fluorescence microscopy, GFP expression was clearly demonstrated at 4 weeks postinjection, primarily in the RPE and the outer retina (Figure 1B-D) in mice treated with 10^8^ or 10^9^ vgc, but not 10^7^ or 10^6^ vgc, of the *AAV-GFP* vector, whereas animals injected with PBS showed no green fluorescence during the 1 to 6 weeks after subretinal injection. GFP expression was maximal at 4 weeks.

Analogous results were obtained by subretinal injection with the AAV vectors expressing VEGF-B167 and 186. Immunofluorescence performed with the mouse monoclonal antibody reacting with both isoforms of mouse VEGF-B at 4 weeks after injection showed that expression of VEGF-B167 (Figure 1E) or VEGF-B186 protein (Figure 1F) was greater in retinas of *AAV-VEGF-B* injected mice, mainly in the RPE, outer segments (OS), outer plexiform layer (OPL), inner nuclear layer (INL), inner plexiform layer (IPL), ganglion cell layer (GCL), and endothelial cells than in mice from the GFP control group (Figure 1G). Data was not shown for the other time points. Real-time PCR assay further confirmed that the mRNA expression levels of VEGF-B167 (Figure 1H) and 186 (Figure 1I) in treated groups were significantly higher (5.8 fold and 12-fold respectively) than in the *AAV-GFP* control group. Administration of 10^8^ vgc or less of *AAV-VEGF-B167* or *186* did not result in increased expression, probably because this was a subthreshold level or because the increase in expression was too small for our assay to detect. Our data for GFP expression was similar to those of previous reports [74,75]; therefore, the same dosage of 10^9^ vgc/μl of AAV vectors was used for all ensuing experiments.

### Neither VEGF-B167 nor 186 induced an obvious inflammatory response

**Figure 2:**
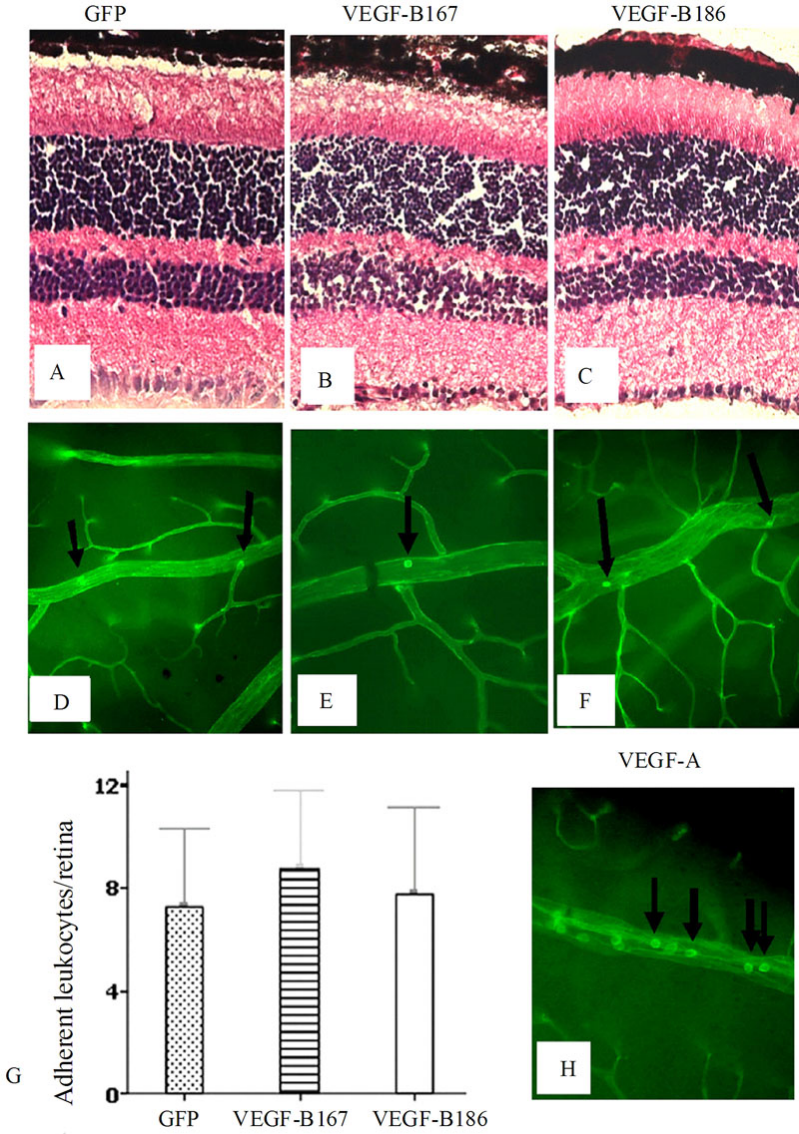
No obvious induction by vascular endothelial growth factor (VEGF)-B167 or 186 of inflammatory response 4 weeks after subretinal injection of adeno-associated virus (*AAV*)-*VEGF-B167* and *186* in C57BL/6J mice. **A**–**C**: Hematoxylin and eosin staining; **D**–**G**: Leukostasis assay indicated that neither *AAV-VEGF-B167* (**E**, **G**) nor *AAV-VEGF-B186* (**F**, **G**) caused significant retinal leukostasis compared to the *AAV-GFP* control (**D**). Data are presented as mean±SEM. Sample sizes for GFP and VEGF-B167 are 10 and for VEGF-B186 is 8. For GFP versus VEGF-B167, p=0.13; for GFP versus VEGF-B186, p=0.3. **H**: An image for comparison showing vascular endothelial growth factor (VEGF)-A-induced leukostasis 6 h after intravitreal administration of 1×10^−6^ M VEGF-A. Arrows are pointing to leukocytes adhering to the vessel wall. Original magnification was ×20.

Based on published references and our own studies, VEGF-A notably induced not only neovascularization, but also an inflammatory response and vasopermeability [68,76-78]. Therefore, we questioned whether intraocular overexpression of both isoforms of VEGF-B would induce similar inflammatory effects. Hematoxylin and eosin staining did not show evidence of inflammatory cell infiltration or edema on retinal sections from the control and experimental groups (Figure 2A-C) and the retinas and their vasculature had relatively normal configuration.

To determine more definitively the inflammatory response to VEGF-B167 and 186, a leukostasis assay was performed and the numbers of leukocytes adherent to retinal vessels were counted. Four weeks after subretinal injection of AAV-vectors, neither *AAV-VEGF-B167* nor *AAV-VEGF-B186* caused significant leukostasis compared to *AAV-GFP* controls (*AAV-VEGF-B167*: 8.8±0.9 adherent leukocytes/retina, n=10; *AAV-VEGF-B186*: 7.75±1.2 adherent leukocytes/retina, n=8; *AAV-GFP*: 7.3±0.9 adherent leukocytes/retina, n=10; P_VEGF-B167/GFP_=0.14; P_VEGF-B186/GFP_=0.38) (Figure 2D-G). A comparative image showing VEGF-A-induced retinal leukostasis is shown in Figure 2H.

### Overexpression of VEGF-B186, but not VEGF-B167, significantly increased vasopermeability of the retina

**Figure 3:**
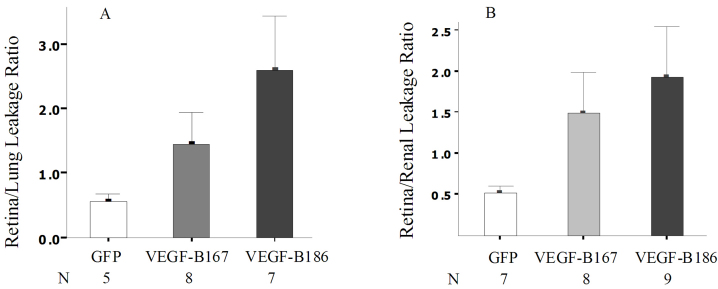
Increased vasopermeability of retinas from B186 but not from B167 vascular endothelial growth factor. Quantitative blood brain barrier (BRB) assays were performed as described in Methods following subretinal injection of *AAV-VEGF-B167*, *AAV-VEGF-B186*, or *AAV*-green fluorescent protein (*GFP*) control vectors in C57BL/6J mice. The retina to lung leakage ratio is shown in **A**; the retina to renal leakage ratio is shown in **B**. Each bar represents the mean (±SEM) ratio of CPM per milligram of retina to CPM per milligram of lung or kidney. Sample sizes for **A**: GFP is n=5; VEGF-B167 is n=8; VEGF-B186 is n=7. **B**: GFP is n=7; VEGF-B167 is n=8; VEGF-B186 is n=9. P-values for the retina to lung leakage ratio relative to GFP were 0.10 for VEGF-B167 and 0.04 for VEGF-B186. P-values for the retina to renal leakage ratio relative to GFP were 0.05 for VEGF-B167 and 0.03 for VEGF-B186, determined by unpaired *t* test, from the GFP control retinas.

Our previous experiments have demonstrated that determining the amount of [3H]-mannitol that enters the retina from the blood, relative to the amount that enters from the lung or kidney, is a sensitive and reproducible technique for quantifying the breakdown of the BRB [55]. Mice treated with a subretinal injection of *AAV-VEGF-B167* showed that the RLLR (Figure 3A) and RRLR (Figure 3B) were 1.44±0.5 (n=8) and 1.48±0.5 (n=8), respectively, which was almost a threefold increase. This was not statistically different compared to those ratios from the *AAV-GFP* control mice (RLLR: 0.55±0.13, n=5; RRLR: 0.52±0.09, n=7; P_RLLR_=0.10; P_RRLR_=0.05). However, mice treated with *AAV-VEGF-B186* showed an RLLR or RRLR of 2.58±0.86 (n=7) or 1.92±0.61 (n=9), respectively, about a four- to fivefold increase that was statistically different compared to those ratios from the *AAV-GFP* control mice (P_RLLR_=0.04; P_RRLR_=0.03).

### Both VEGF-B167 and VEGF-B186 increased choroidal neovascularization

**Figure 4:**
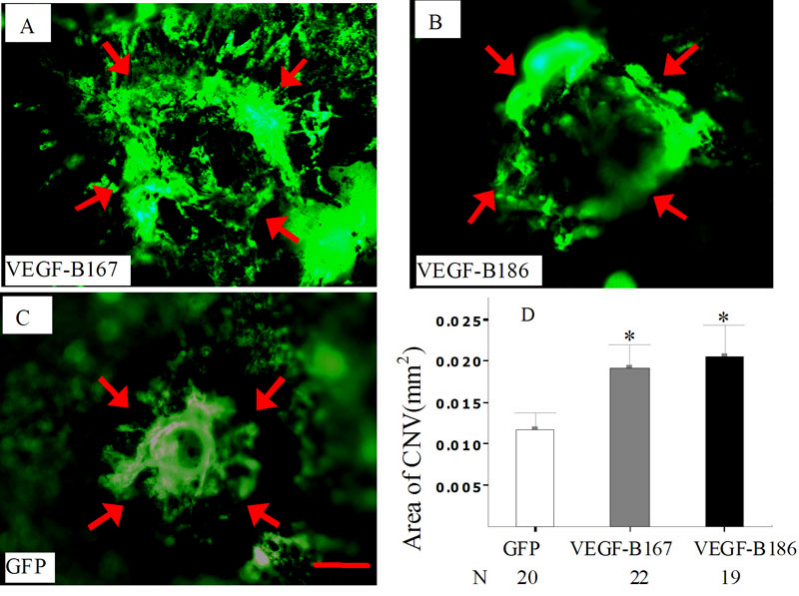
Increased choroidal neovascularization caused by overexpression of adeno-associated virus-vascular endothelial growth factor (*AAV-VEGF*)*-B167* and *186* at rupture sites in Bruch’s membrane. **A**–**C**: Two weeks after subretinal injection of *AAV-VEGF-B167* **(A)**, *AAV-VEGF-B186* **(B)**, or *AAV-GFP* **(C)**, mice underwent laser photocoagulation that ruptured Bruch’s membrane in three locations in each eye. Fourteen days later, mice were perfused with fluorescein-labeled dextran, and choroidal flatmounts were prepared and examined by fluorescence microscopy. Compared to eyes treated with a subretinal injection of *AAV-GFP* **(C)**, the area of choroidal neovascularization at Bruch’s membrane rupture sites was significantly increased in eyes given a subretinal injection of *AAV-VEGF-B167* (**A**, **D**) or *186* (**B**, **D**). The arrows define the limits of the choroidal neovascularization. **D** shows that the area of choroidal neovascularization (CNV), expressed in mm^2^, is significantly increased following a subretinal injection of *AAV-VEGFB-B167* or *186*. Data are expressed as mena±SEM. Statistical comparisons were made by unpaired *t* test (*p<0.05). Images **A** to **C** have the same scale. Scale bar represents 100 μm.

To determine whether the overexpression of VEGF-B would provide any increase in NV, we compared the *AAV-VEGF-B167*, *AAV-VEGF-B186*, and *AAV-GFP*-treated eyes in an experimental model of CNV. Four weeks after vector injection and two weeks after rupture of Bruch’s membrane with laser photocoagulation, the area of CNV at Bruch’s membrane rupture sites appeared larger in eyes that had been given subretinal injections of *AAV-VEGF-B167* or *AAV-VEGF-B186* (Figure 4A,B) when compared with those given a subretinal injection of *AAV-GFP* (Figure 4C). Measurement of the area of CNV at rupture sites by image analysis, with the investigator masked with respect to treatment group, confirmed that the mean area of CNV was significantly greater in eyes given subretinal injections of either *AAV-VEGF-B167* (0.019±0.003 mm^2^, n=22, p=0.02) or *AAV-VEGF-B186* (0.021±0.017 mm^2^, n=19, p=0.02) (Figure 4D) when compared with eyes given *AAV-GFP* (0.012±0.002 mm^2^, n=20) by the corresponding route of administration.

### Both VEGF-B167 and VEGF-B186 increased retinal neovascularization in oxygen induced ischemic retinopathy

**Figure 5:**
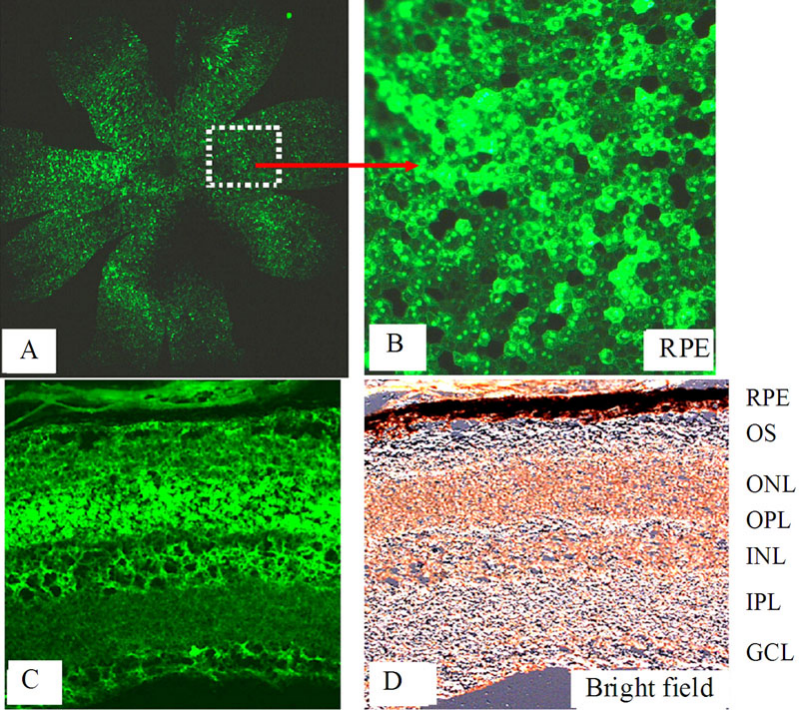
Representative images show transgene expression after intravitreous delivery of recombinant adeno-associated virus-green fluorescent protein (*AAV-GFP*) in the oxygen induced ischemic retinopathy (OIR) model. At postnatal day 7, pups received an intravitreal injection of *AAV-GFP* vector and were then placed in a 75% oxygen box for 5 days. Thereafter, pups were returned to room air for another 5 days. At day 10 after injection (postnatal day 17), pups were killed. Choroidal flatmounts (**A**, **B**) and cryosections (**C**, **D**) were prepared. Green fluorescent protein expression was directly observed using fluorescence microscopy. Original magnifications were as follows: **A** ×5, **B**: ×40, **C**, **D**: ×20. The figure shows increased expression of the reporter gene, *GFP*. The following layers are illustrated: RPE: retinal pigmental epithelium, OS: outer segment, OPL: outer plexiform layer, INL: inner nuclear layer, IPL: inner plexiform layer, GCL: ganglion cell layer.

**Figure 6:**
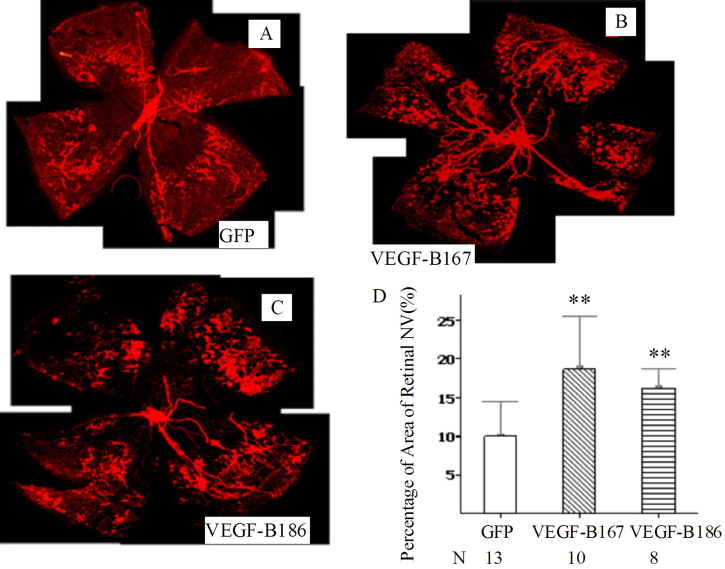
Both vascular endothelial growth factor-B167 and 186 increased retinal neovascularization in oxygen induced ischemic retinopathy. At postnatal day 7 (P7), pups received an intravitreal injection of *AAV-VEGF-B167*, *AAV-VEGF-B186*, or *AAV-GFP* vectors, then were placed in a 75% oxygen box for 5 days and returned to room air at P12 for another 5 days. At P17, mice were sacrificed, retinal-whole mounts prepared, and retinal neovascularization quantified as described in Methods. Images from **A** to **C** are representative retina whole mounts from mice that received *AAV-GFP* (**A**), *AAV-VEGF-B167* (**B**), or *AAV-VEGF-B186* (**C**). Original magnification was ×5. All images were taken under the same conditions for optimal comparison. **D:** Statistical analysis indicated that both isoforms of VEGF-B significantly increased the retinal neovascularization area compared with the GFP control group. The graph shows that *AAV-VEGF-B167* and *186* promote increased retinal neovascularization in the oxygen induced ischemic retinopathy model. Data are expressed as mena±SEM.

Given that overexpression of VEGF-B167 and 186 significantly increased the area of CNV, we next explored whether they would increase the area of pathological retinal NV. Developing retinal NV in the mouse model of OIR has proven reliable and quantifiable over 17 days, and is widely used to clarify the molecular changes in neovascular eye diseases and to screen anti-angiogenic compounds [79-81]. At P7, pups received an intravitreal injection of *AAV-VEGF-B167*, *AAV-VEGF-B186*, or *AAV-GFP* vectors and were then placed in a 75% oxygen box for 5 days. Thereafter, pups were returned to room air for another 5 days. At day 3, 6, 8, and 10 after vector delivery, three *AAV GFP*-injected pups were killed to observe the GFP protein expression, which was initially and focally seen in the retina at day 3, and became stronger and more diffuse at day 6 and 10 (Figure 5). Therefore, protein expression of the GFP reporter gene at the above time points indicated that *AAV VEGF-B167* or *186* could also effectively be expressed at these time points. At P17 (10 days after receiving the AAV vectors), increased expression of VEGF-B was also confirmed in *AAV-VEGF-B*-injected pups by quantitative PCR. At P17, mice treated with *AAV-VEGF-B167* (18.6%±2.1%, n=10, p<0.001) or *AAV-VEGF-B186* (16.2%±0.89%, n=8, p=0.001) developed more extensive retinal NV than control mice treated with *AAV-GFP* (9.97% ± 1.3%, n=13; Figure 6A-D).

## Discussion

Overexpression of VEGF-B can be achieved using an AAV vector, and the source of the gene product is widespread in the retina. Both intravitreal and subretinal administration of the vector were effective, with subretinal administration leading to the greatest increase in VEGF-B expression in adult mice. In adult mice, intravitreal administration was not as effective as subretinal delivery (data not shown). That is why we chose subretinal delivery in adult mice. However, in pups, whose retinas are still developing and whose inner limiting membranes are not yet well developed, the vector can easily penetrate the entire retina from the vitreous; therefore, intravitreal administration of the vectors was implemented, since it yielded the same expression profile and was technically less difficult than subretinal injection in neonates. Previous attempts have been made using an adenovirus vector, which resulted in phenotypic changes such as retinal folding and occasional CNV, but these effects were largely due to an inflammatory response caused by the adenovirus vector. Inflammation was eliminated by using the AAV vector, so that the true effect of VEGF-B could be evaluated.

The functions of VEGF-B in normal and pathological conditions are poorly understood, but since it binds to VEGFR1, as does PlGF, it was thought that these activities might be similar to those of PlGF. AAV vector constructs were generated to investigate whether VEGF-B promoted pathological angiogenesis and inflammation, as did PlGF [22,29,32], and whether its overexpression elicited any phenotypic changes in the retina. The vasoproliferative and vasopermeability activities of VEGF have generally been attributed to VEGFR2, but the finding that anti-PlGF inhibits pathological angiogenesis [22] shows that VEGFR1 also plays a role. The present study shows that overexpression of VEGF-B enhances retinal NV resulting from ischemia and CNV following laser treatment, providing further evidence that VEGFR1 plays a role and suggesting that PlGF and VEGF-B share signaling mechanisms. The finding that VEGF-B is highly expressed in the CNV area, 7 days after laser treatment, and that anti-VEGF-B reduces CNV, supports the conclusions about its role in CNV formation and its potential as a target molecule [58].

Previous studies have reported apparently contradictory results on the angiogenic properties of VEGF-B, with findings varying from VEGF-B having no angiogenic effect at all [42,51,53] to its promotion of unrestricted angiogenesis [47], its ability to potentiate rather than induce angiogenesis when it is transgenically expressed in endothelial cells [49], or its ability to induce selective revascularization of the ischemic myocardium but not the revascularization of other organs [54,55]. We have previously reported that when VEGF-B is injected into the nonischemic myocardium, it elicits only a modest angiogenic response consisting of the generation of enlarged vessels devoid of α-SMA-positive cells [56,57]. Our work here demonstrates that both of the VEGF-B isoforms exert a significant angiogenic effect under conditions of ocular damage, potentiating pathologic blood vessel formation in animal models of CNV and retinal NV. Previous studies that failed to show an angiogenic effect of VEGF-B evaluated different systems, used a different type of vector (adenovirus), or administered the protein, rather than the gene. It appears clear that the retina and heart respond differently to the overexpression of VEGF-B. The adenovirus vector is proinflammatory, and the influence of inflammatory cells may alter the effect of VEGF-B. It could also be that continuous exposure to elevated VEGF-B levels achieved by gene transduction, rather than by intermittent increases from injections of the protein, is necessary for revealing VEGF-B’s angiogenic effect in the retina and choroid. This possibly explains why the present study’s results differ from those of earlier studies in which the absence of an angiogenic effect was observed following the injections of VEGF-B protein [58]. Zhang et al. [58] showed that blocking VEGF-B resulted in the inhibition of CNV and retinal NV. The present study clearly shows that increased expression of VEGF-B enhances choroidal and retinal NV.

We also showed that VEGF-B186 promotes vascular permeability, confirming the role of VEGFR1 in this activity as well. The finding that VEGF-B167 does not significantly increase vascular permeability, although our results do appear to show a trend in that direction, is likely due to the greater capacity of VEGF-B186 to diffuse through the retinal tissue due to the absence of the heparin-binding moiety. With a greater sample size, VEGFB-167 will also possibly demonstrate a significant increase in vascular permeability, but this effect is enhanced by the 186 isoform. The pro-inflammatory activity of VEGF has generally been associated with VEGFR1, but VEGF-B does not appear to be pro-inflammatory, even though anti-PlGF inhibits inflammatory infiltration into CNV lesions [22]. This observation further supports the conclusion that VEGF-B and PlGF activate VEGFR1 in a subtly different manner, possibly through the use of different coreceptors.

The present study gives insight into the isolated effect of VEGF-B on the retina and choroid in the absence of disease-associated stimuli or stimuli that normally cause VEGF upregulation. In DR, neovascular AMD, and retinal vasculitis, VEGF upregulation is seen with inflammation, and if an intravitreal VEGF inhibitor is administered, the inflammation is reduced. This leads to the suspicion that VEGF is a primary inflammatory mediator in these inflammatory disorders and that VEGF may simply be a mediator that compromises the BRB, allowing cytokines, antibodies, complement, and leukocytes to leave the blood stream if there is the appropriate stimulus for doing so [82,83]. The present study demonstrates that, if there is no primary cause of inflammation, then the opening of the BRB will not, by itself, cause inflammation.

Upregulation of VEGF-B, to our knowledge, has not been clinically demonstrated in ocular disease, but it has in renal cell carcinoma [84]. The finding that VEGF-B promotes retinal and choroidal NV and BRB breakdown suggests that VEGF-B may contribute to the complications of vasoproliferative ocular disorders and that it could be a therapeutic target for treating angiogenic ocular disorders and disorders leading to macular edema.

## References

[1] Ruiz de Almodovar C Lambrechts D Mazzone M Carmeliet P Role and therapeutic potential of VEGF in the nervous system. Physiol Rev 2009 89 607 48 19342615 10.1152/physrev.00031.2008

[2] Witmer AN Blaauwgeers HG Weich HA Alitalo K Vrensen GF Schlingemann RO Altered expression patterns of VEGF receptors in human diabetic retina and in experimental VEGF-induced retinopathy in monkey. Invest Ophthalmol Vis Sci 2002 43 849 57 11867607

[3] Ogawa S Oku A Sawano A Yamaguchi S Yazaki Y Shibuya M A novel type of vascular endothelial growth factor, VEGF-E (NZ-7 VEGF), preferentially utilizes KDR/Flk-1 receptor and carries a potent mitotic activity without heparin-binding domain. J Biol Chem 1998 273 31273 82 9813035 10.1074/jbc.273.47.31273

[4] Meyer M Clauss M Lepple-Wienhues A Augustin HG Waltenberger J Ziche M Lanz C Büttner M Rziha HJ Dehio C A novel vascular endothelial growth factor encoded by Orf virus, VEGF-E, mediates angiogenesis via signalling through VEGFR-2 (KDR) but not VEGFR-1 (Flt-1) receptor tyrosine kinases. EMBO J 1999 15 363 74 9889193 10.1093/emboj/18.2.363 PMC1171131

[5] Joukov V Pajusola K Kaipainen A Chilov D Lahtinen I Kukk E Saksela O Kalkkinen N Alitalo K A novel vascular endothelial growth factor, VEGF-C, is a ligand for the Flt4 (VEGFR-3) and KDR (VEGFR-2) receptor tyrosine kinases. EMBO J 1996 15 290 8 8617204 PMC449944

[6] Achen MG Jeltsch M Kukk E Mäkinen T Vitali A Wilks AF Alitalo K Stacker SA Vascular endothelial growth factor D (VEGF-D) is a ligand for the tyrosine kinases VEGF receptor 2 (Flk1) and VEGF receptor 3 (Flt4). Proc Natl Acad Sci USA 1998 95 548 53 9435229 10.1073/pnas.95.2.548 PMC18457

[7] Witmer AN Vrensen GF Van Noorden CJ Schlingemann RO Vascular endothelial growth factors and angiogenesis in eye disease. Prog Retin Eye Res 2003 22 1 29 12597922 10.1016/s1350-9462(02)00043-5

[8] Witmer AN Dai J Weich HA Vrensen GF Schlingemann RO Expression of vascular endothelial growth factor receptors 1, 2, and 3 in quiescent endothelia. J Histochem Cytochem 2002 50 767 77 12019293 10.1177/002215540205000603

[9] Park JE Chen HH Winer J Houck KA Ferrara N Placenta growth factor. Potentiation of vascular endothelial growth factor bioactivity, in vitro and in vivo, and high affinity binding to Flt-1 but not to Flk-1/KDR. J Biol Chem 1994 269 25646 54 7929268

[10] Cao R Xue Y Hedlund EM Zhong Z Tritsaris K Tondelli B Lucchini F Zhu Z Dissing S Cao Y VEGFR1-mediated pericyte ablation links VEGF and PlGF to cancer-associated retinopathy. Proc Natl Acad Sci USA 2010 107 856 61 20080765 10.1073/pnas.0911661107 PMC2818941

[11] Murata T Nakagawa K Khalil A Ishibashi T Inomata H Sueishi K The relation between expression of vascular endothelial growth factor and breakdown of the blood-retinal barrier in diabetic rat retinas. Lab Invest 1996 74 819 25 8606491

[12] Miller JW Adamis AP Shima DT Yeo T-K Yeo K-T D'Amore PA Moulton RS O'Reilly MS Folkman J Dvorak HF Brown LF Berse B Vascular endothelial growth factor/vascular permeability factor is temporally and spatially correlated with ocular angiogenesis in a primate model. Am J Pathol 1994 145 574 84 7521577 PMC1890317

[13] Tolentino MJ Miller JW Gragoudas ES Jakobiec FA Flynn E Chatzistefanou K Ferrara N Adamis AP Intravitreous injections of vascular endothelial growth factor produce retinal ischemia and microangiopathy in an adult primate. Ophthalmology 1996 103 1820 8 8942877 10.1016/s0161-6420(96)30420-x

[14] Duh E Aiello LP Vascular endothelial growth factor and diabetes: the agonist versus antagonist paradox. Diabetes 1999 48 1899 906 10512352 10.2337/diabetes.48.10.1899

[15] Schlingemann RO van Hinsbergh VW Role of vascular permeability factor/vascular endothelial growth factor in eye disease. Br J Ophthalmol 1997 81 501 12 9274417 10.1136/bjo.81.6.501 PMC1722234

[16] Kim I Ryan AM Rohan R Amano S Agular S Miller JW Adamis AP Constitutive expression of VEGF, VEGFR-1, and VEGFR-2 in normal eyes. Invest Ophthalmol Vis Sci 1999 40 2115 21 10440268

[17] Gilbert RE Vranes D Berka JL Kelly DJ Cox A Wu LL Stacker SA Cooper ME Vascular endothelial growth factor and its receptors in control and diabetic rat eyes. Lab Invest 1998 78 1017 27 9714188

[18] Stitt AW Simpson DA Boocock C Gardiner TA Murphy GM Archer DB Expression of vascular endothelial growth factor (VEGF) and its receptors is regulated in eyes with intra-ocular tumours. J Pathol 1998 186 306 12 10211121 10.1002/(SICI)1096-9896(1998110)186:3<306::AID-PATH183>3.0.CO;2-B

[19] Smith G McLeod D Foreman D Boulton M Immunolocalisation of the VEGF receptors FLT-1, KDR, and FLT-4 in diabetic retinopathy. Br J Ophthalmol 1999 83 486 94 10434875 10.1136/bjo.83.4.486 PMC1722996

[20] Saint-Geniez M Kurihara T Seklyama E Maldonado AE D’Amore PA An essential role for RPE-derived soluble VEGF in the maintenance of the choriocapillaris. Proc Natl Acad Sci USA 2009 106 18751 6 19841260 10.1073/pnas.0905010106 PMC2774033

[21] Ponticelli S Marasco D Tarallo V Albuquerque RJ Mitola S Takeda A Stassen JM Presta M Ambati J Ruvo M De Falco S Modulation of angiogenesis by a tetrameric tripeptide that antagonizes vascular endothelial growth factor receptor 1. J Biol Chem 2008 283 34250 9 18922791 10.1074/jbc.M806607200 PMC2590698

[22] Van de Veire S Stalmans I Heindryckx F Oura H Tijeras-Raballand A Schmidt T Loges S Albrecht I Jonckx B Vinckier S Van Steenkiste C Tugues S Rolny C De Mol M Dettori D Hainaud P Coenegrachts L Contreres JO Van Bergen T Cuervo H Xiao WH Le Henaff C Buysschaert I Kharabi Masouleh B Geerts A Schomber T Bonnin P Lambert V Haustraete J Zacchigna S Rakic JM Jiménez W Noël A Giacca M Colle I Foidart JM Tobelem G Morales-Ruiz M Vilar J Maxwell P Vinores SA Carmeliet G Dewerchin M Claesson-Welsh L Dupuy E Van Vlierberghe H Christofori G Mazzone M Detmar M Collen D Carmeliet P Further pharmacological and genetic evidence for the efficacy of PlGF inhibition in cancer and eye disease. Cell 2010 141 178 90 20371353 10.1016/j.cell.2010.02.039

[23] Kliche S Waltenberger J VEGF receptor signaling and endothelial function. IUBMB Life 2001 52 61 6 11795595 10.1080/15216540252774784

[24] Hiratsuka S Minowa O Kuno J Noda T Shibuya M Flt-1 lacking the tyrosine kinase domain is sufficient for normal development and angiogenesis in mice. Proc Natl Acad Sci USA 1998 95 9349 54 9689083 10.1073/pnas.95.16.9349 PMC21341

[25] Ambati BK Nozaki M Singh N Takeda A Jani PD Suthar T Albuquerque RJC Richter E Sakurai E Newcomb MT Kleinman ME Caldwell RB Lin Q Ogura Y Orecchia A Samuelson DA Agnew DW St. Leger J Green WR Mahasreshti PJ Curiel DT Kwan D Marsh H Ikeda S Leiper LJ Collinson JM Bogdanovich S Khurana TS Shibuya M Baldwin ME Ferrara N Gerber H-P De Falco S Witta J Baffi JZ Raisler BJ Ambati J Corneal avascularity is due to soluble VEGF receptor-1. Nature 2006 443 993 7 17051153 10.1038/nature05249 PMC2656128

[26] Chappell JC Taylor SM Ferrara N Bautch VL Local guidance of emerging vessel sprouts requires soluble Flt-1. Dev Cell 2009 17 377 86 19758562 10.1016/j.devcel.2009.07.011 PMC2747120

[27] Pavlakovic H Becker J Albuquerque R Wilting J Ambati J Soluble VEGFR-2: an antilymphangiogenic variant of VEGF receptors. Ann N Y Acad Sci 2010 Suppl 1 E7 15 20961309 10.1111/j.1749-6632.2010.05714.x PMC3062194

[28] Ziche M Morbidelli L Choudhuri R Zhang HT Donnini S Granger HJ Bicknell R Nitric oxide synthase lies downstream from vascular endothelial growth factor-induced but not basic fibroblast growth factor-induced angiogenesis. J Clin Invest 1997 99 2625 34 9169492 10.1172/JCI119451 PMC508108

[29] Luttun A Tjwa M Moons L Wu Y Angelillo-Scherrer A Liao F Nagy JA Hooper A Priller J De Klerck B Compernolle V Daci E Bohlen P Dewerchin M Herbert JM Fava R Matthys P Carmeliet G Collen D Dvorak HF Hicklin DJ Carmeliet P Revascularization of ischemic tissues by PlGF treatment, and inhibition of tumor angiogenesis, arthritis and atherosclerosis by anti-Flt1. Nat Med 2002 8 831 40 12091877 10.1038/nm731

[30] Carmeliet P Moons L Luttun A Vincenti V Compernolle V De Mol M Wu Y Bono F Devy L Beck H Scholz D Acker T DiPalma T Dewerchin M Noel A Stalmans I Barra A Blacher S Vandendriessche T Ponten A Eriksson U Plate KH Foidart JM Schaper W Charnock-Jones DS Hicklin DJ Herbert JM Collen D Persico MG Synergism between vascular endothelial growth factor and placental growth factor contributes to angiogenesis and plasma extravasation in pathological conditions. Nat Med 2001 7 575 83 11329059 10.1038/87904

[31] Fischer C Jonckx B Mazzone M Zacchigna S Loges S Pattarini L Chorianopoulos E Liesenborghs L Koch M De Mol M Autiero M Wyns S Plaisance S Moons L van Rooijen N Giacca M Stassen JM Dewerchin M Collen D Carmeliet P Anti-PlGF inhibits growth of VEGF(R)-inhibitor-resistant tumors without affecting healthy vessels. Cell 2007 131 463 75 17981115 10.1016/j.cell.2007.08.038

[32] Fischer C Mazzone M Jonckx B Carmeliet P FLT1 and its ligands VEGFB and PlGF: drug targets for anti-antiogenic therapy? Natl Rev 2008 8 942 56 10.1038/nrc2524 19029957

[33] Monsky WL Fukumura D Gohongi T Ancukiewcz M Weich HA Torchilin VP Yuan F Jain RK Augmentation of transvascular transport of macromolecules and nanoparticles in tumors using vascular endothelial growth factor. Cancer Res 1999 59 4129 35 10463618

[34] Odorisio T Schietroma C Zaccaria ML Cianfarani F Tiveron C Tatangelo L Failla CM Zambruno G Mice overexpressing placenta growth factor exhibit increased vascularization and vessel permeability. J Cell Sci 2002 115 2559 67 12045226 10.1242/jcs.115.12.2559

[35] Clauss M Weich H Breier G Knies U Röckl W Waltenberger J Risau W The vascular endothelial growth factor receptor Flt-1 mediates biological activities. Implications for a functional role of placenta growth factor in monocyte activation and chemotaxis. J Biol Chem 1996 271 17629 34 8663424 10.1074/jbc.271.30.17629

[36] Sawano A Iwai S Sakurai Y Ito M Shitara K Nakahata T Shibuya M Flt-1, vascular endothelial growth factor receptor 1, is a novel cell surface marker for the lineage of monocyte-macrophages in humans. Blood 2001 97 785 91 11157498 10.1182/blood.v97.3.785

[37] Enholm B Paavonen K Ristimäki A Kumar V Gunji Y Klefstrom J Kivinen L Laiho M Olofsson B Joukov V Eriksson U Alitalo K Comparison of VEGF, VEGF-B, VEGF-C and Ang-1 mRNA regulation by serum, growth factors, oncoproteins and hypoxia. Oncogene 1997 14 2475 83 9188862 10.1038/sj.onc.1201090

[38] Rakic JM Lambert V Devy L Luttun A Carmeliet P Claes C Nguyen L Foidart JM Noël A Munaut C Placental growth factor, a member of the VEGF family, contributes to the development of choroidal neovascularization. Invest Ophthalmol Vis Sci 2003 44 3186 93 12824270 10.1167/iovs.02-1092

[39] Vinores SA Xiao WH Aslam S Shen J Oshima Y Nambu H Liu H Carmeliet P Campochiaro PA Implication of the hypoxia response element of the Vegf promoter in mouse models of retinal and choroidal neovascularization, but not retinal vascular development. J Cell Physiol 2006 206 749 58 16245301 10.1002/jcp.20525

[40] Olofsson B Jeltsch M Eriksson U Alitalo K Current biology of VEGF-B and VEGF-C. Curr Opin Biotechnol 1999 10 528 35 10600689 10.1016/s0958-1669(99)00024-5

[41] Nash AD Baca M Wright C Scotney PD The biology of vascular endothelial growth factor-B (VEGF-B). Pulm Pharmacol Ther 2006 19 61 9 16286239 10.1016/j.pupt.2005.02.007

[42] Malik AK Baldwin ME Peale F Fuh G Liang WC Lowman H Meng G Ferrara N Gerber HP Redundant roles of VEGF-B and PlGF during selective VEGF-A blockade in mice. Blood 2006 107 550 7 16189273 10.1182/blood-2005-05-2047

[43] Bellomo D Headrick JP Silins GU Paterson CA Thomas PS Gartside M Mould A Cahill MM Tonks ID Grimmond SM Townson S Wells C Little M Cummings MC Hayward NK Kay GF Mice lacking the vascular endothelial growth factor-B gene (Vegfb) have smaller hearts, dysfunctional coronary vasculature, and impaired recovery from cardiac ischemia. Circ Res 2000 86 E29 35 10666423 10.1161/01.res.86.2.e29

[44] Aase K von Euler G Li X Pontén A Thorén P Cao R Cao Y Olofsson B Gebre-Medhin S Pekny M Alitalo K Betsholtz C Eriksson U Vascular endothelial growth factor-B-deficient mice display an atrial conduction defect. Circulation 2001 104 358 64 11457758 10.1161/01.cir.104.3.358

[45] Grimmond S Lagercrantz J Drinkwater C Silins G Townson S Pollock P Gotley D Carson E Rakar S Nordenskjöld M Ward L Hayward N Weber G Cloning and characterization of a novel human gene related to vascular endothelial growth factor. Genome Res 1996 6 124 31 8919691 10.1101/gr.6.2.124

[46] Olofsson B Pajusola K von Euler G Chilov D Alitalo K Eriksson U Genomic organization of the mouse and human genes for vascular endothelial growth factor B (VEGF-B) and characterization of a second splice isoform. J Biol Chem 1996 271 19310 7 8702615 10.1074/jbc.271.32.19310

[47] Silvestre JS Tamarat R Ebrahimian TG Le-Roux A Clergue M Emmanuel F Duriez M Schwartz B Branellec D Lévy BI Vascular endothelial growth factor-B promotes in vivo angiogenesis. Circ Res 2003 93 114 23 12805240 10.1161/01.RES.0000081594.21764.44

[48] Wright CE Effects of vascular endothelial growth factor (VEGF)A and VEGFB gene transfer on vascular reserve in a conscious rabbit hindlimb ischaemia model. Clin Exp Pharmacol Physiol 2002 29 1035 9 12366398 10.1046/j.1440-1681.2002.03773.x

[49] Mould AW Greco SA Cahill MM Tonks ID Bellomo D Patterson C Zournazi A Nash A Scotney P Hayward NK Kay GF Transgenic overexpression of vascular endothelial growth factor-B isoforms by endothelial cells potentiates postnatal vessel growth in vivo and in vitro. Circ Res 2005 97 e60 70 16109918 10.1161/01.RES.0000182631.33638.77

[50] Yoon YS Losordo DW All in the family: VEGF-B joins the ranks of proangiogenic cytokines. Circ Res 2003 93 87 90 12881472 10.1161/01.RES.0000084992.10766.36

[51] Reichelt M Shi S Hayes M Kay G Batch J Gole GA Browning J Vascular endothelial growth factor-B and retinal vascular development in the mouse. Clin Experiment Ophthalmol 2003 31 61 5 12580897 10.1046/j.1442-9071.2003.00602.x

[52] Rissanen TT Markkanen JE Gruchala M Heikura T Puranen A Kettunen MI Kholová I Kauppinen RA Achen MG Stacker SA Alitalo K Ylä-Herttuala S VEGF-D is the strongest angiogenic and lymphangiogenic effector among VEGFs delivered into skeletal muscle via adenoviruses. Circ Res 2003 92 1098 106 12714562 10.1161/01.RES.0000073584.46059.E3

[53] Bhardwaj S Roy H Gruchala M Viita H Kholova I Kokina I Achen MG Stacker SA Hedman M Alitalo K Ylä-Herttuala S Angiogenic responses of vascular endothelial growth factors in periadventitial tissue. Hum Gene Ther 2003 14 1451 62 14577925 10.1089/104303403769211664

[54] Li X Tjwa M Van Hove I Neven E Paavonen K Jeltsch M Juan TD Sievers RE Chorianopoulos E Wada H Vanwildemeersch M Noel A Foidart JM Springer ML von Degenfeld G Dewerchin M Blau HM Alitalo K Eriksson U Carmeliet P Moons L Enholm B Reevaluation of the role of VEGF-B suggests a restricted role in the revascularization of the ischemic myocardium. Arterioscler Thromb Vasc Biol 2008 28 1614 20 18511699 10.1161/ATVBAHA.107.158725 PMC2753879

[55] Lähteenvuo JE Lähteenvuo MT Kivelä A Falkevall A Klar J Heikura T Rissanen TT Vähäkangas E Korpisalo P Enholm B Carmeliet P Alitalo K Eriksson U Ylä-Herttuala S Rosenlew C Vascular endothelial growth factor-B induces myocardium-specific angiogenesis and arteriogenesis via vascular endothelial growth factor receptor-1- and neuropilin receptor-1-dependent mechanisms. Circulation 2009 119 845 56 19188502 10.1161/CIRCULATIONAHA.108.816454

[56] Zentilin L Puligadda U Lionetti V Zacchigna S Collesi C Pattarini L Ruozi G Camporesi S Sinagra G Pepe M Recchia FA Giacca M Cardiomyocyte VEGFR-1 activation by VEGF-B induces compensatory hypertrophy and preserves cardiac function after myocardial infarction. FASEB J 2010 24 1467 78 20019242 10.1096/fj.09-143180

[57] Pepe M Mamdani M Zentilin L Csiszar A Qanud K Zacchigna S Ungvari Z Puligadda U Moimas S Xu X Edwards JG Hintze TH Giacca M Recchia FA Intramyocardial VEGF-B167 Gene Delivery Delays the Progression Towards Congestive Failure in Dogs With Pacing-Induced Dilated Cardiomyopathy. Circ Res 2010 106 1893 903 20431055 10.1161/CIRCRESAHA.110.220855 PMC4879815

[58] Zhang F Tang Z Hou X Lennartsson J Li Y Koch AW Scotney P Lee C Arjunan P Dong L Kumar A Rissanen TT Wang B Nagai N Fons P Fariss R Zhang Y Wawrousek E Tansey G Raber J Fong GH Ding H Greenberg DA Becker KG Herbert JM Nash A Yla-Herttuala S Cao Y Watts RJ Li X VEGF-B is dispensable for blood vessel growth but critical for their survival, and VEGF-B targeting inhibits pathological angiogenesis. Proc Natl Acad Sci USA 2009 106 6152 7 19369214 10.1073/pnas.0813061106 PMC2669337

[59] Inagaki K Fuess S Storm TA Gibson GA Mctiernan CF Kay MA Nakai H Robust systemic transduction with AAV9 vectors in mice: efficient global cardiac gene transfer superior to that of AAV8. Mol Ther 2006 14 45 53 16713360 10.1016/j.ymthe.2006.03.014 PMC1564441

[60] Rabinowitz JE Rolling F Li C Xiao X Samulski RJ Conrath H Xiao W Cross-packaging of a single adeno-associated virus (AAV) type 2 vector genome into multiple AAV serotypes enables transduction with broad specificity. J Virol 2002 76 791 801 11752169 10.1128/JVI.76.2.791-801.2002 PMC136844

[61] Arsic N Zentilin L Zacchigna S Santoro D Stanta G Salvi A Sinagra G Giacca M Induction of functional neovascularization by combined VEGF and angiopoietin-1 gene transfer using AAV vectors. Mol Ther 2003 7 450 9 12727107 10.1016/s1525-0016(03)00034-0

[62] Zacchigna S Pattarini L Zentilin L Moimas S Carrer A Sinigaglia M Arsic N Tafuro S Sinagra G Giacca M Bone marrow cells recruited through the neuropilin-1 receptor promote arterial formation at the sites of adult neoangiogenesis in mice. J Clin Invest 2008 118 2062 75 18483621 10.1172/JCI32832 PMC2381745

[63] Allocca M Mussolino C Garcia-Hoyos M Sanges D Iodice C Petrillo M Vandenberghe LM Wilson JM Marigo V Surace EM Auricchio. Novel adeno-associated virus serotypes efficiently transduce murine photoreceptors. J Virol 2007 81 11372 80 17699581 10.1128/JVI.01327-07 PMC2045569

[64] Lebherz C Maguire A Tang W Bennett J Wilson JM Novel AAV serotypes for improved ocular gene transfer. J Gene Med 2008 10 375 82 18278824 10.1002/jgm.1126 PMC2842078

[65] Lei B Zhang K Yue Y Ghosh A Duan D Adeno-associated virus serotype-9 efficiently transduces the retinal outer plexiform layer. Mol Vis 2009 15 1374 82 19626133 PMC2713732

[66] Lei B Zhang K Yue Y Ghosh A Duan D Adeno-associated virus serotype-9 mediated retinal outer plexiform layer transduction is mainly through the photoreceptors. Adv Exp Med Biol 2010 664 671 8 20238072 10.1007/978-1-4419-1399-9_77

[67] Mori K Duh E Gehlbach P Ando A Takahashi K Pearlman J Mori K Yang HS Zack DJ Ettyreddy D Brough DE Wei LL Campochiaro PA Pigment epithelium-derived factor inhibits retinal and choroidal neovascularization. J Cell Physiol 2001 188 253 63 11424092 10.1002/jcp.1114

[68] Vinores SA Xiao WH Shen J Campochiaro PA TNF-alpha is critical for ischemia-induced leukostasis, but not retinal neovascularization nor VEGF-induced leakage. J Neuroimmunol 2007 182 73 9 17107717 10.1016/j.jneuroim.2006.09.015 PMC1800833

[69] Derevjanik NL Vinores SA Xiao WH Mori K Turon T Hudish T Dong S Campochiaro PA Quantitative assessment of the integrity of the blood-retinal barrier in mice. Invest Ophthalmol Vis Sci 2002 43 2462 7 12091451

[70] Chen J Connor KM Aderman CM Smith LE Erythropoietin deficiency decreases vascular stability in mice. J Clin Invest 2008 118 526 33 18219389 10.1172/JCI33813 PMC2213374

[71] Connor KM Krah NM Dennison RJ Aderman CM Chen J Guerin KI Sapieha P Stahl A Willett KL Smith LE Quantification of oxygen-induced retinopathy in the mouse: a model of vessel loss, vessel regrowth and pathological angiogenesis. Nat Protoc 2009 4 1565 73 19816419 10.1038/nprot.2009.187 PMC3731997

[72] Tobe T Ortega S Luna JD Ozaki H Okamoto N Derevjanik NL Vinores SA Basilico C Campochiaro PA Targeted disruption of the FGF2 gene does not prevent choroidal neovascularization in a murine model. Am J Pathol 1998 153 1641 6 9811357 10.1016/S0002-9440(10)65753-7 PMC1853405

[73] Smith LEH Wesolowski E McLellan A Kostyk SK D'Amato R Sullivan R D'Amore PA Oxygen-induced retinopathy in the mouse. Invest Ophthalmol Vis Sci 1994 35 101 11 7507904

[74] Allocca M Mussolino C Garcia-Hoyos M Sanges D Iodice C Petrillo M Vandenberghe LH Wilson JM Marigo V Surace EM Auricchio A Novel adeno-associated virus serotypes efficiently transduce murine photoreceptors. J Virol 2007 81 11372 80 17699581 10.1128/JVI.01327-07 PMC2045569

[75] Auricchio A Rolling F Adeno-associated viral vectors for retinal gene transfer and treatment of retinal diseases. Curr Gene Ther 2005 5 339 48 15975011 10.2174/1566523054065020

[76] Angelo LS Kurzrock R Vascular endothelial growth factor and its relationship to inflammatory mediators. Clin Cancer Res 2007 13 2825 30 17504979 10.1158/1078-0432.CCR-06-2416

[77] Kataru RP Jung K Jang C Yang H Schwendener RA Baik JE Han SH Alitalo K Koh GY Critical role of CD11b+ macrophages and VEGF in inflammatory lymphangiogenesis, antigen clearance, and inflammation resolution. Blood 2009 113 5650 9 19346498 10.1182/blood-2008-09-176776

[78] Joussen AM Murata T Tsujikawa A Kirchhof B Bursell SE Adamis AP Leukocyte-mediated endothelial cell injury and death in the diabetic retina. Am J Pathol 2001 158 147 52 11141487 10.1016/S0002-9440(10)63952-1 PMC1850259

[79] Mammoto A Connor KM Mammoto T Yung CW Huh D Aderman CM Mostoslavsky G Smith LE Ingber DE A mechanosensitive transcriptional mechanism that controls angiogenesis. Nature 2009 457 1103 8 19242469 10.1038/nature07765 PMC2708674

[80] Hellström M Phng LK Hofmann JJ Wallgard E Coultas L Lindblom P Alva J Nilsson AK Karlsson L Gaiano N Yoon K Rossant J Iruela-Arispe ML Kalén M Gerhardt H Betsholtz C Dll4 signalling through Notch1 regulates formation of tip cells during angiogenesis. Nature 2007 445 776 80 17259973 10.1038/nature05571

[81] Kubota Y Hirashima M Kishi K Stewart CL Suda T Leukemia inhibitory factor regulates microvessel density by modulating oxygen-dependent VEGF expression in mice. J Clin Invest 2008 118 2393 403 18521186 10.1172/JCI34882 PMC2398738

[82] Bressler SB Introduction: Understanding the role of angiogenesis and antiangiogenic agents in age-related macular degeneration. Ophthalmology 2009 116 supplement S1 7 19800534 10.1016/j.ophtha.2009.06.045

[83] Cao J Zhao L Li Y Liu Y Xiao W Song Y Huang D Yancopoulos GD Wiegand SJ Wen R A subretinal matrigel rat choroidal neovascularization (CNV) model and inhibition of CNV and associated inflammation and fibrosis by VEGF trap. Invest Ophthalmol Vis Sci 2010 51 6009 17 20538989 10.1167/iovs.09-4956 PMC3061520

[84] Gunningham SP Currie MJ Han C Turner K Scott PA Robinson BA Harris AL Fox SB Vascular endothelial growth factor-B and vascular growth factor-C expression in renal cell carcinomas: regulation by the von Hippel-Lindau gene and hypoxia. Cancer Res 2001 61 3206 11 11306510

